# Joint Impacts of Drought and Habitat Fragmentation on Native Bee Assemblages in a California Biodiversity Hotspot

**DOI:** 10.3390/insects12020135

**Published:** 2021-02-05

**Authors:** Keng-Lou James Hung, Sara S. Sandoval, John S. Ascher, David A. Holway

**Affiliations:** 1 Section of Ecology, Behavior and Evolution, Division of Biological Sciences, University of California—San Diego, 9500 Gilman Drive, La Jolla, CA 92037, USA; ssandoval@csusm.edu (S.S.S.); dholway@ucsd.edu (D.A.H.); 2 Department of Ecology and Evolutionary Biology, University of Toronto, 25 Willcocks Street, Toronto, ON M5S 3B2, Canada; 3 Department of Biological Sciences, National University of Singapore, 14 Science Drive 4, Singapore 117543, Singapore; dbsajs@nus.edu.sg

**Keywords:** coastal sage scrub, global climate change, habitat loss and fragmentation, pollinators, California drought, *LasioglossumDialictus*

## Abstract

**Simple Summary:**

Global climate change is causing more frequent and severe droughts, which can have serious impacts on our environment. To examine how a severe drought in 2014 impacted wild bees in scrub habitats of San Diego, California, we compared bee samples collected before and after the drought. We also investigated whether habitat loss and fragmentation worsened the impacts of drought on wild bees by comparing samples collected from large natural reserves to those from small fragments of scrub habitat embedded in urban areas. Samples collected after the drought contained fewer bee species and fewer individual bees of most species, indicating that bee populations suffered losses during the drought. However, after-drought samples contained large numbers of *Dialictus* sweat bees, indicating that some bee species benefitted from environmental conditions present during the drought. The impact of drought on the composition of bee samples was three fold higher than the impact of habitat fragmentation, and habitat fragmentation did not appear to have exacerbated the impacts of drought. Our findings highlight the importance of studying how impacts of climate change compare with impacts of habitat loss and other threats to biodiversity conservation.

**Abstract:**

Global climate change is causing more frequent and severe droughts, which could have serious repercussions for the maintenance of biodiversity. Here, we compare native bee assemblages collected via bowl traps before and after a severe drought event in 2014 in San Diego, California, and examine the relative magnitude of impacts from drought in fragmented habitat patches versus unfragmented natural reserves. Bee richness and diversity were higher in assemblages surveyed before the drought compared to those surveyed after the drought. However, bees belonging to the *Lasioglossum* subgenus *Dialictus* increased in abundance after the drought, driving increased representation by small-bodied, primitively eusocial, and generalist bees in post-drought assemblages. Conversely, among non-*Dialictus* bees, post-drought years were associated with decreased abundance and reduced representation by eusocial species. Drought effects were consistently greater in reserves, which supported more bee species, than in fragments, suggesting that fragmentation either had redundant impacts with drought, or ameliorated effects of drought by enhancing bees’ access to floral resources in irrigated urban environments. Shifts in assemblage composition associated with drought were three times greater compared to those associated with habitat fragmentation, highlighting the importance of understanding the impacts of large-scale climatic events relative to those associated with land use change.

## 1. Introduction

Climate change is considered one of the leading threats to biodiversity worldwide [1,2,3], with impacts associated with both climate warming and increased frequency and severity of extreme climate events [4], such as droughts. Although droughts are a natural feature in many ecosystems worldwide [5,6], the severity, frequency, and duration of recent drought episodes associated with anthropogenic climate change may have adverse effects, even in ecosystems that experience regular seasonal droughts [7,8,9]. Droughts may impact biological communities both directly by imposing abiotic stressors [7,10] and indirectly by altering interactions among organisms experiencing such abiotic stress [11,12].

Plant-pollinator mutualisms are an example of a key biological interaction that may be vulnerable to the effects of drought (reviewed in [13]). During drought events, annual plants may remain dormant in the seed bank, while perennial plants may reduce their reproductive investment by producing fewer flowers [9,14]. Even if flowers do bloom, reduced nectar and pollen production caused by drought could result in the breakdown of interactions among plant and pollinator mutualists [14,15]. Although a number of studies have investigated impacts of drought on specific plants and their suite of pollinators [9,15,16], as well as on entire plant assemblages [10,17], fewer studies have examined the impacts of drought on pollinator assemblages [18,19]. Further, to our knowledge, no published study to date has investigated the joint impacts of drought and other anthropogenic stressors such as habitat loss and habitat intensification on pollinator assemblages.

Here, we report on a dataset of native bees (Hymenoptera: Anthophila), collected from endangered coastal sage scrub habitats in San Diego County, California, that fortuitously spanned a severe drought event in the year 2014 [7,20]. Native bee surveys were conducted during two consecutive years before the drought event and two consecutive years after the event in the same series of study sites. Although no survey was conducted during the drought event, comparisons of native bee assemblages immediately before and after the drought event provide insight into how drought may have modified the assemblages. As the dataset was collected in a series of scrub habitat fragments and large natural reserves in order to examine the responses of bee assemblages to habitat loss and fragmentation [21,22], it also allows us to investigate the joint impacts of drought and habitat fragmentation on bee assemblages.

Using this dataset, we test the hypothesis that the reduction of floral resources during the drought event negatively impacts the survival and reproduction of native bees. Accordingly, we predict that observed bee richness and abundance would be lower in the years immediately following the drought, compared to the years before the drought, as a result of local extirpations and population declines. Additionally, we also hypothesize that bee species may be differentially impacted by drought as a function of their life history traits. Of the many axes of life history variation exhibited by bees in our system, we focus our inquiry on three traits that may be drivers of bee species’ responses to drought.

First, bee species vary with respect to their diet breadth, where some species specialize on the pollen of a narrow set of plant species or families, while others utilize pollen from numerous botanical families. Generalist (i.e., polylectic) species tend to be less susceptible to environmental perturbations in general [23,24,25], likely in part due to their ability to find suitable food resources regardless of turnovers in plant assemblages resulting from disturbance. During droughts, polylectic species may be able to survive and reproduce so long as some subset of the plant assemblage continues to bloom. On the other hand, pollen specialist (i.e., oligolectic and mesolectic) species may exhibit enhanced synchronicity with their host plants’ blooms [26], and in xeric regions such as ours, they may have a greater tendency to evolve multi-year diapause strategies to closely track their hosts so as to avoid emerging in years when adverse climatic conditions prohibit their host plants from blooming [19].

Second, bee species vary with respect to their degree of sociality. While the majority of species in our system are solitary in the broad sense (i.e., non-eusocial), several genera consist at least in part of primitively eusocial species, which form annual colonies founded by a single queen produced by the previous generation. Eusocial species may perform poorly relative to solitary species in years of reduced resource availability because of their higher sensitivity to resource gaps during their long colony lifetime [27,28]. However, all eusocial species in our system exhibit a generalist diet, which, as discussed above, may allow them to profit from those floral resources that are available during the drought. Their division of labor and ability to coordinate foraging efforts [29,30] may also translate into greater foraging success when resources are scant and patchily distributed.

Third, bee species in our system span at least an order of magnitude in body length [22]. Given the known correlation between bee body size and mobility [31], larger bees may be better able to access flowers growing in drought refugia (e.g., riparian corridors, north-facing slopes, runoff zones of impervious surfaces) and could more quickly recolonize areas where populations were extirpated by drought. On the other hand, smaller bees require less resources to successfully reproduce, and are perhaps less likely to become locally extirpated as a result.

As the above discussion demonstrates, life history traits have the potential to either enhance or reduce bee species’ ability to withstand droughts. Thus, understanding how the potential tradeoffs of each life history trait translate into realized population dynamics in drought scenarios necessitates examining empirical, assemblage-wide data. Empirical examination is especially important in systems such as ours, which may include both xeric-adapted and mesic-adapted bee species (based on known life history traits and distributions of the species; see [22]), which may differ in their capacity to respond to drought. If life history traits indeed influence the response of bee species to drought, we expect to detect differences in the species composition of assemblages before versus after drought. In an extreme case, there should also be a homogenization of bee faunas after drought as bee assemblages become dominated by a subset of species that possess combinations of traits that confer enhanced persistence during drought.

The impacts of drought on our system may not act in isolation, as drought has been known to interact with other forms of anthropogenic perturbations to produce synergistic impacts on ecosystems and biological communities [32,33]. In our case, habitat fragmentation may potentially exacerbate or ameliorate the impacts of drought. On one hand, because bee assemblages in our habitat fragments are taxonomically and functionally depauperate compared to those in relatively intact natural reserves [22], they may be missing many of the taxa that can withstand, avoid (i.e., via extended diapause), or quickly recover from drought (e.g., xeric-adapted species). Also, habitat fragments may fail to encompass drought refugia due to their small size, and their isolation may hinder the recolonization [34] of bees from drought refugia once drought has ended. On the other hand, the urban landscape may itself provide unique refugia from drought in the form of irrigated plantings [35,36] and flowering weeds that thrive in the runoff zones of paved roads [37]. These urban refugia may lessen the impact of drought on bees persisting in fragments, at least for the subset of taxa that can access and utilize the food resources therein. Understanding how drought and habitat fragmentation interact to impact native bee assemblages will be important in guiding efforts to conserve biodiversity and ecosystem function under continuing habitat loss and increased climatic variability. From a more general ecological perspective, examining how taxonomically and functionally depauperate bee assemblages in fragments respond to drought is also a useful test of the theory that the biodiversity of a community is correlated with its robustness to perturbations [38,39].

## 2. Materials and Methods

### 2.1. Study System

Data collection occurred in coastal sage scrub (CSS) habitats in San Diego County, CA, USA. San Diego, along with the rest of southern California, is a global biodiversity hotspot for both vascular plants [40] and native bees [41,42], and experiences a Mediterranean climate, with the majority of rainfall occurring in the winter months. The CSS flora is adapted to drought conditions [7], as with plant communities in other Mediterranean-climate ecosystems; nevertheless, the CSS flora negatively responds both to severe one-year droughts and to persistent, multi-year droughts [7,43]. The majority of CSS has succumbed to urbanization, agriculture, and other land-use modifications; less than 15% of its original extent in California remains intact [44]. Furthermore, anthropogenic landscapes (i.e., urban and agricultural areas) fragment much of the remaining CSS. Loss and fragmentation of CSS has detrimental effects on a variety of animal taxa [22,34,45,46,47].

### 2.2. Data Collection

**Figure 1:**
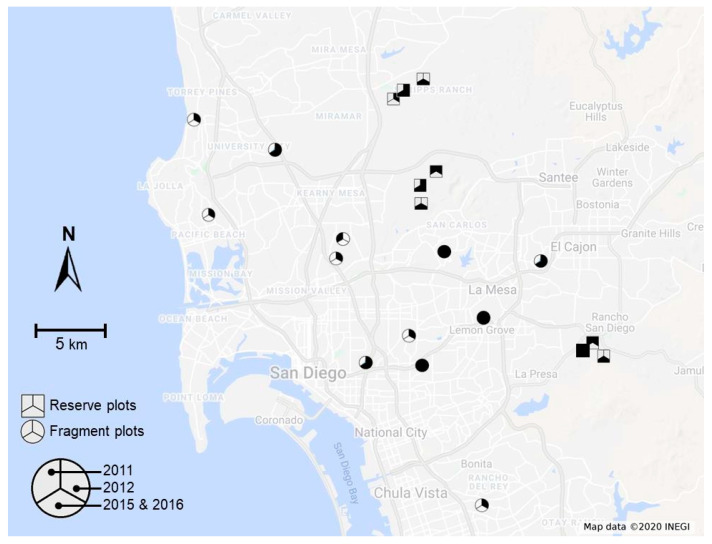
Map of the study region showing the location of 1-ha study plots in large scrub reserves (squares) and scrub fragments (circles). Within each symbol, filling of distinct sectors indicates the year(s) in which the plot was sampled.

**Figure 2:**
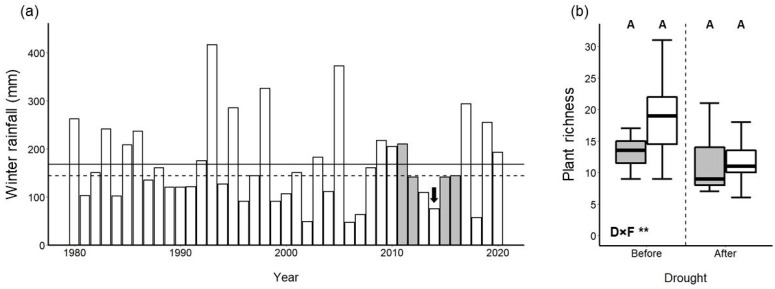
Biotic and abiotic context of native bee surveys. Bar plot (**a**) depicts rainfall in the San Diego region accumulated through the winter months (Nov–Feb inclusive) preceding the spring of each calendar year, with years of bee surveys depicted in gray bars. The black arrow depicts the severe drought event of 2014; solid and dotted horizontal lines depict 40-year mean and median winter rainfall in the period of 1980–2020, respectively. Box plot (**b**) depicts plot-level species richness of entomophilous plants that bloomed in scrub reserve plots (gray boxes) and scrub fragment plots (white boxes) during the timeframe of our surveys. Boxes show central 50% of data; bold horizontal lines represent the median; whiskers extend from the quartiles to 1.5× the interquartile range (or most extreme values of data, whichever is closest to median). Different letters above the boxes indicate treatment combinations that are statistically distinct from one another (*p* < 0.05) in post-hoc pairwise tests. Statistical significance of the interaction between drought status and fragmentation status (“D×F”) is provided: ** *p* ≤ 0.01.

Bee assemblage data considered in this study were collected as part of a series of previous studies investigating the effects of habitat fragmentation on native bee diversity [21,22]. We collected data in 1-ha study plots located either in fragments of CSS (internal area 2.7 ha–117 ha, median = 30 ha; see Table S1) embedded in urban (mostly residential) landscapes or in large, relatively undisturbed CSS reserves (internal area >> 500 ha), most of which are protected from development (Figure 1). Data were collected in 2011, 2012, 2015, and 2016, with our entire study period encompassed within the 2011–2017 California drought event [48]. Although all data were collected during the prolonged, region-wide drought, our study years were ones with relatively ample winter rainfall (November–February) in San Diego (Figure 2a, data obtained from the National Weather Service at www.weather.gov) that preceded and immediately followed the single worst year of drought in 2014 [7,20]. Not all study plots were sampled in all years (see Table S1) because our specific goals differed over time, but each study year had, at a minimum, n = 4 study plots in fragments (mean = 6.75 plots across all years) and n = 4 study plots in reserves (mean = 5.5 plots across all years). Although only a subset of study plots were sampled both before and after the drought (3 reserve plots and 6 fragment plots, see Table S1), retaining only this subset of plots in analyses yielded results that were qualitatively similar to those of analyses that used the full dataset (see Figures S1 and S2).

During the peak blooming season of CSS flora (March–August), we deployed colored bowl traps to collect native bees in our study plots. We generally sampled bees at 2–3 plots per day and on 3–4 days per week as weather conditions permitted, with the average sampling frequency each year (mean = 13 d in 2011, 26 d in 2012, 15 d in 2015, 19 d in 2016) depending largely on the number of plots sampled, additional data collection goals, and availability of field assistants. Bowl traps consisted of bright white, 96-mL plastic bowls 7 cm in diameter, painted fluorescent blue or yellow or left unpainted, and filled with 60 mL of unscented detergent solution. Bowl traps were deployed from ca. 0800 h to ca. 1600 h on each survey day, in roughly linear transects containing 15 bowls each (5 of each color), with nearest bowls placed 5–10 m apart. In 2011 and 2012, we deployed two bowl transects (i.e., 30 bowls) per study plot during each survey, whereas in 2015 and 2016, we deployed a single bowl transect (i.e., 15 bowls) per study plot during each survey. Although bowl traps are known to exhibit bias with respect to the composition and relative abundance of bee species sampled (reviewed and summarized by [49,50]), this sampling method allows for standardized sampling of multiple sites simultaneously and can minimize human biases in capture rate among sites [51]. We expect that our use of bowl traps resulted in a biased sample of the bee assemblages occurring in our plots. However, since all study plots in all study years shared the same bowl trap design and deployment methodology (all deployed by K.-L.J.H.), they should also share the same, consistent bias with respect to the identities of the taxa that were overrepresented and underrepresented in their samples. As a result, we believe that the relative abundances of bees we collected should reflect at least the activity of the subset of the native bee fauna amenable to sampling by bowl traps in our area. To further minimize systematic bias resulting from positive or negative covariance of bowl trap capture rates and the availability of floral resources in the surrounding landscape (reviewed by [49,50]), we limited our analyses to data collected from April–June of each study year. In each of our four study years, annual and perennial plants bloomed in abundance during all three months; CSS bee assemblages also exhibit their greatest species richness during the period from April–June in our study region [52].

All native bees collected in the bowl traps (n = 11,055 individuals) were washed, dried, mounted, labeled, identified, and databased. The vast majority of individuals (n = 10,969) were identified in previous studies [21,22] to one of 167 species or morphospecies (hereafter referred to collectively as “species”), although a small subset of male morphospecies (n = 86 individuals) could not be confidently matched to female morphospecies and were excluded from analyses requiring species-level taxonomic resolution. For each species, we used published literature and phylogenetic inference (for details, see [22]) to assign three life history traits that we hypothesized would be important in determining how bees respond to drought: body size (measured as intertegular distance, see [53]), diet breadth (polylectic versus oligolectic and mesolectic species), and sociality (non-eusocial species versus primitively eusocial taxa including *Bombus*, *Augochlorella*, *Halictus*, and many *Lasioglossum* species, in particular species of subgenus *Dialictus*). Cleptoparasitic species, which invade the nests of pollen-collecting species and lay their eggs therein such that their larvae usurp the nest provisions, were assigned a unique category for both diet breadth and sociality. Nearly all eusocial bee species are polylectic [29], including all eusocial species in our system, such that the two traits are not statistically independent. However, since eusocial species constituted only 43% of the polylectic species in our dataset (34 out of 80 species), we chose to examine these two life history traits separately despite the strong correlation between them, with the caveat that differences in diet breadth may be a major contributor to any differences in the response of eusocial versus non-eusocial species. The western honey bee (*Apis mellifera* L.), which was the numerically dominant bee species in our system [21] and the only species that exhibits advanced eusociality, is not native to North America and was thus not considered in our analyses. Table S2 lists the species and their associated life history traits. Specimens were deposited at the San Diego Natural History Museum (SDMC) and the University of California, San Diego Pollinator Collection managed by the Holway Laboratory.

Bee assemblages are strongly influenced by the local composition and blooming phenology of plant species [54,55]. Thus, on the same days that we surveyed bees, we also documented the identities of the plant species (both native and non-native) in bloom within the boundaries of the 1-ha study plots. Plant species that were present, but not blooming, were not counted in these surveys.

### 2.3. Statistical Analyses

We performed two sets of analyses to investigate additive and interactive impacts of drought and habitat fragmentation on the studied bee assemblages. We used univariate analyses to examine how diversity, abundance, and proportional representation of different functional traits differed across bee assemblages. We used multivariate analyses to examine the joint impacts of drought and habitat fragmentation on bee assemblage composition. We performed all analyses using program R, version 3.6.3 [56].

Univariate analyses: We constructed generalized linear mixed models (GLMMs) using the *lme4* package [57] to examine the joint impacts of drought and habitat fragmentation on bee abundance, species richness, Shannon-Wiener diversity (*H*), body size (approximated using intertegular distance), and the proportional representation of individuals belonging to polylectic species (i.e., pollen generalists) and primitively eusocial species. To account for the fact that the number of survey days and the number of individuals collected varied across study plots and study years, we estimated species richness and Shannon-Wiener diversity by interpolating or extrapolating assemblages [58] to a common sample size equal to the median abundance among all study plots in all years (n = 227 individuals). Species richness and diversity estimations were performed with the *iNEXT* package [59], which uses models described in Colwell et al. [58] to generate interpolated and extrapolated estimates.

All GLMMs include fragmentation status (scrub fragments versus unfragmented reserves), drought status (before (2011–2012) versus after (2015–2016)), and their interaction as independent variables; study plot identity and study year (the latter nested within drought status) were random-intercept terms. The locations of two study plots in CSS reserves were shifted slightly between 2012 and 2015 (see Table S1); however, given the small displacements (plot centers moved <300 m in each case) relative to the overall dispersion of study plots in the landscape (Figure 1), we chose to treat pre- and post-displacement locations as the same study plot in our analyses. Our choice to construct models using drought status as a main effect and study year as a random term reflects our goal of detecting impacts of drought in isolation from interannual variation well known in bee assemblages [60]. Although this model structure does not enable explicitly examining potential trajectories of recovery from drought (i.e., by comparing dataset of 2015 to that of 2016), the short-term recovery of bee faunas is beyond the scope of this manuscript because of the lack of longer-term data (e.g., from 2017 and 2018) that would have otherwise enabled distinguishing true recovery from interannual variation [60]. Further, we note that auxiliary analyses that used study year instead of drought status as a main effect failed to detect consistent evidence of recovery in our bee faunas between 2015 and 2016 (see Figure S3). We used the *lmerTest* package [61] to determine the significance of interactions between drought status and fragmentation status, and used the *emmeans* package [62] to perform post-hoc pairwise comparisons between all possible combinations of fragmentation status and drought status, with Tukey’s adjustment for multiple comparisons.

We analyzed blooming plant species richness (i.e., the richness of plant species that were in bloom during our surveys) pooled across all sampling dates in each plot within each year using a Gaussian GLMM (link = identity). We analyzed bee abundance using a Poisson GLMM (link = log), with the number of transect-days (i.e., number of transects deployed per day × number of days surveyed) included as an offset variable such that the model effectively examines differences in numbers of bees collected per transect-day. We analyzed species richness, Shannon-Wiener diversity, and average body size using Gaussian GLMMs. The proportional representation of individuals belonging to eusocial species and to polylectic species were analyzed using binomial GLMMs (link = logit), which use the ratio of the numbers of individuals belonging to the target versus non-target state (i.e., eusocial versus solitary, polylectic versus non-polylectic) as the dependent variable.

Upon evaluating the results of our models for body size and proportional representation of eusocial and polylectic species, we decided to construct an additional binomial GLMM to examine the proportional representation of individuals belonging to *Lasioglossum* subgenus *Dialictus* (Halictidae), which accounted for the majority of individuals belonging to species that are small-bodied, polylectic, and eusocial. We also constructed a Poisson GLMM to examine *Dialictus* abundance. Further, to investigate the extent to which this group may have driven our other univariate results, we repeated our analyses that examined bee abundance, body size, diet breadth, and sociality after excluding all *Dialictus* individuals from our dataset. These additional models for non-*Dialictus* bee assemblages were structured identically to their counterparts constructed using the full dataset.

Multivariate analyses: We performed a permutational multivariate analysis of variance (i.e., PERMANOVA; [63]) and permutational tests of multivariate dispersion (i.e., PERMDISP; [64,65]), where PERMANOVA addressed whether or not the species composition of bee assemblages differed across groupings, while PERMDISP addressed whether or not the compositional dispersion of bee assemblages (i.e., beta diversity) differed across groupings. Both statistical procedures were based on a matrix of Bray-Curtis dissimilarity scores between bee assemblages in all possible pairs of plot-year combinations. We performed PERMANOVA using the “adonis” function and PERMDISP using the “permutest.betadisper” function, both in the *vegan* package [66]. To control for the fact that assemblages differed in the number of individuals sampled, we standardized all assemblages by dividing the abundance of each species by the assemblage total, such that Bray-Curtis scores were influenced only by the identities and relative abundances of species, and not the total abundance of each assemblage.

As there is currently no mixed-effects framework available for PERMDISP and PERMANOVA, we used study year rather than drought status as an independent variable in these analyses. Since PERMDISP does not accommodate the inclusion of multiple independent variables, we performed three distinct analyses to examine whether or not the beta diversity of bee assemblages differed across (i) levels of fragmentation status, (ii) study years, and (iii) treatment combinations (i.e., eight combinations between two levels of fragmentation status and four years). PERMANOVA, on the other hand, does accommodate multiple independent variables, so we included fragmentation status, year, and their interaction in this analysis. We used the *pairwiseAdonis* package [67] to perform post-hoc comparisons between each pair of study years, with a Benjamini-Hochberg false discovery rate correction to account for multiple comparisons.

## 3. Results

### 3.1. Univariate Analyses

**Figure 3:**
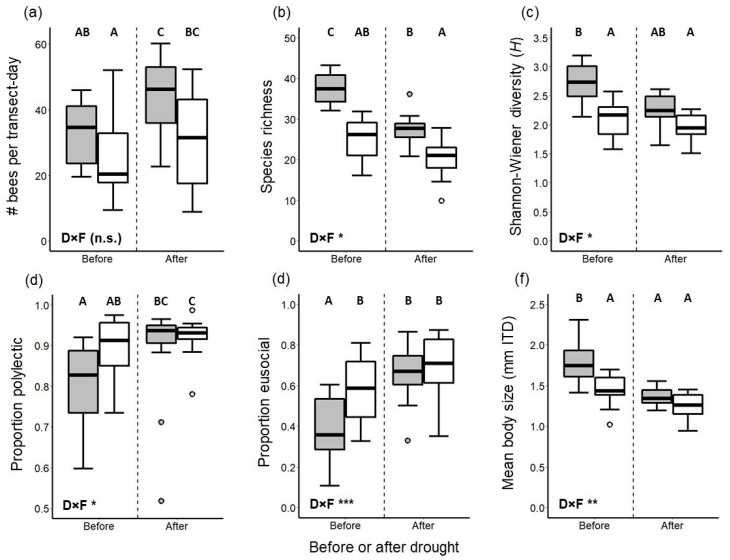
Comparisons of bee assemblages in scrub reserve plots (gray boxes) and scrub fragment plots (white boxes) before (2011 and 2012) and after (2015 and 2016) a severe drought event in 2014. Panels (**a**–**f**) depict different characteristics of bee assemblages; in panel (**f**), “ITD” stands for intertegular distance. Boxplots are as in Figure 2b; circles indicate outliers, if any. “D×F” denotes the statistical significance of the interaction between drought status and fragmentation status: n.s. *p* > 0.05, * *p* ≤ 0.05, ** *p* ≤ 0.01, *** *p* ≤ 0.001.

Drought and habitat fragmentation had both additive and interactive impacts on bee assemblages in our coastal sage scrub study system despite having comparatively modest impacts on the richness of entomophilous plants in bloom. Plot-level species richness of plants in bloom did not differ between any pair of treatment combinations (i.e., combinations of reserves versus fragments and before versus after drought); however, there was a significant interaction between drought status and fragmentation status (Gaussian GLMM *t* = 3.21, *p* = 0.003) (Figure 2b). In contrast to our prediction, bee abundance was higher post-drought than pre-drought for both reserves and fragments (Figure 3a) and exhibited no significant drought-by-fragmentation interaction (*p* > 0.05). On the other hand, bee species richness was reduced post-drought relative to pre-drought as predicted (Figure 3b), although only in reserves and not in fragments (drought-by-fragmentation interaction: Gaussian GLMM *t* = 2.01, *p* = 0.050). Bee diversity exhibited the same trend as species richness (drought-by-fragmentation interaction: Gaussian GLMM *t* = 2.45, *p* = 0.019), except that the reduction of diversity post-drought in reserves was only marginally significant (Figure 3c, Table S3).

With respect to the impacts of drought and habitat fragmentation on the life history trait distributions of bee assemblages, the proportional representation of individuals belonging to polylectic species was higher post-drought than pre-drought (Figure 3d), with the pre- versus post-drought difference being greater in reserves than in fragments (drought-by-fragmentation interactions: binomial GLMM *z* = 2.33, *p* = 0.020). The proportional representation of individuals belonging to primitively eusocial species increased after drought, (Figure 3e), while bee body size decreased after drought (Figure 3f). In both cases, however, the effect of drought was only observed in reserves and not in fragments (drought-by-fragmentation interactions: Gaussian GLMM *t* = 2.76, *p* = 0.009 for body size; binomial GLMM *z* = 6.08, *p* < 0.0001 for proportion eusocial).

**Figure 4:**
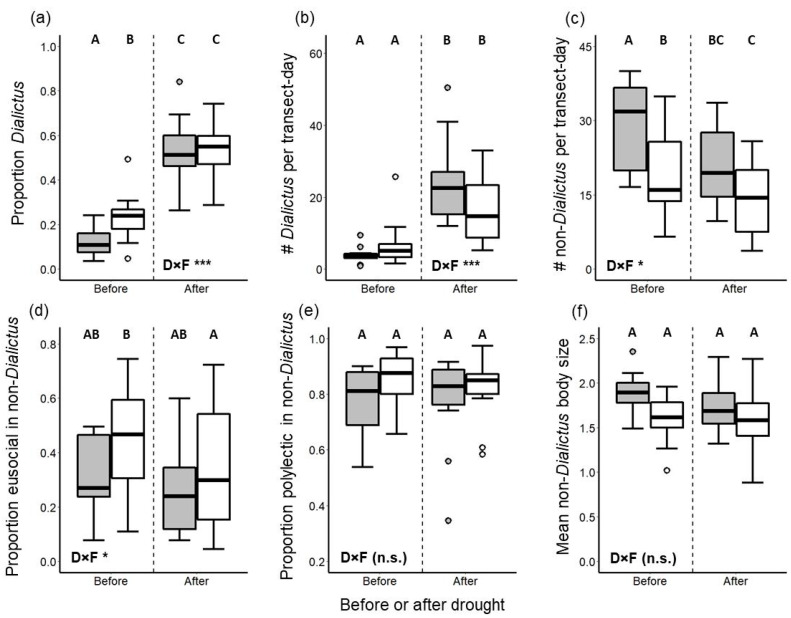
Comparisons of *Dialictus* and non-*Dialictus* bee assemblages in scrub reserve plots (gray boxes) and scrub fragment plots (white boxes) before (2011 and 2012) and after (2015 and 2016) a severe drought event in 2014. Panels (**a**–**f**) depict different characteristics of bee assemblages; in panel (**f**), the metric used for bee body size is mm intertegular distance. Boxplots and denotations of the statistical significance of the interaction between drought status and fragmentation status (“D×F”) are as in Figure 3.

The proportional representation as well as total abundance of individuals belonging to *Lasioglossum* subgenus *Dialictus* (a taxon comprising small-bodied, eusocial, polylectic bees) increased after drought (Figure 4a,b), with the post-drought increases in both being greater in reserves than in fragments (drought-by-fragmentation interaction: binomial GLMM *z* = 6.42, *p* < 0.0001 for proportion *Dialictus*; Poisson GLMM *z* = 6.16, *p* < 0.0001 for *Dialictus* abundance). In contrast, the abundance of non-*Dialictus* bees decreased after the drought in both reserves and fragments (Figure 4c), although, again, this decrease was more pronounced in reserves than in fragments (drought-by-fragmentation interaction: Poisson GLMM *z* = 2.22, *p* = 0.026). The proportional representation of eusocial bees among non-*Dialictus* individuals was lower post-drought only in fragments (Figure 4d) and not in reserves (drought-by-fragmentation interaction: binomial GLMM *z* = 2.28, *p* = 0.023). Lastly, among non-*Dialictus* bees, both the proportional representation of polylectic bees and average body size did not differ between pre- and post-drought for either reserves or fragments (Figure 4e,f). Detailed statistical outputs are reported in Table S3.

### 3.2. Multivariate Analyses

**Figure 5:**
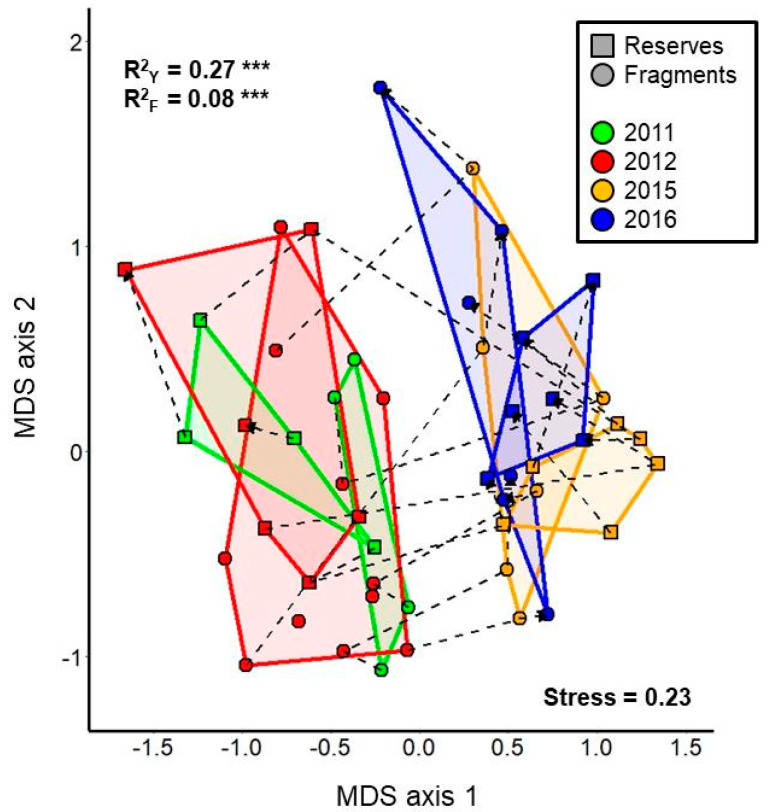
Nonmetric multidimensional scaling (NMDS) ordination plot of bee assemblages in reserve plots (squares) and fragment plots (circles) across four years of sampling. Points depicting sequential years of sampling at the same study plot are connected by dotted lines that terminate in an arrow at the point that depicts the last year of sampling for the plot. The ordination was constructed based on Bray-Curtis dissimilarity scores between all possible pairs of plot-year combinations. Dissimilarity scores were calculated based on relative abundances of bee species after standardizing the total abundance of each assemblage to a sum of 1. Statistical significance and *R*^2^ value are indicated for each PERMANOVA independent variable: Y = study year, F = fragmentation status (reserves versus fragments); *** *p* ≤ 0.001. There was no statistically significant interaction between study year and fragmentation status.

Unlike the results of the univariate analyses, multivariate analyses revealed only additive effects of drought and habitat fragmentation on bee assemblage composition (Figure 5). Although bee assemblage composition differed significantly both across study years (PERMANOVA *F*_3,41_ = 5.98, *p* < 0.001) and between reserves and fragments (*F*_1,41_ = 5.17, *p* < 0.001), there was no significant interaction between the two variables (*F*_3,41_ = 0.66, *p* = 0.92). Based on *R*^2^ values, study year explained more than three times the amount of variation in the data compared to fragmentation status. Post-hoc tests revealed that bee assemblages in study years with the same drought status differed little, if at all, from each other, while pre- versus post-drought comparisons revealed strong differences in bee assemblage composition (Table 1). On the other hand, we found no evidence of differences in compositional dispersion (i.e., beta diversity) between bee assemblages across study years (*F*_3,45_ = 0.84, *p* = 0.50), between reserves and fragments (PERMDISP *F*_1,47_ = 0.024, *p* = 0.87), or across treatment combinations (i.e., combinations of study year and fragmentation status; *F*_7,41_ = 0.91, *p* = 0.52).

## 4. Discussion

Our analyses revealed strong impacts of drought on bee assemblages in the fragmented coastal sage scrub of San Diego. Post-drought bee assemblages had fewer species, were greater in abundance, and were more strongly represented by individuals belonging to eusocial, polylectic, and smaller-bodied species (particularly *Lasioglossum* subgenus *Dialictus*). In every assemblage-level univariate comparison, we observed differences in pre- versus post-drought assemblages in reserves, and often in fragments as well. Dissimilarity-based, multivariate analysis also revealed strong differences in bee species composition between pre- and post-drought assemblages.

The most consistent pattern to emerge from our analysis is that habitat fragmentation appeared to ameliorate, rather than exacerbate, the impact of drought whenever a statistically significant interaction between fragmentation status and drought status was detected. Other studies on native bee assemblages have also found that small habitat fragments embedded in urbanized landscapes possess features that appear beneficial to certain aspects of bee biology [68,69,70]. In our case, this ameliorating effect of fragments may potentially have arisen from the ability of at least some bees in fragments to access blooming floral resources growing in the urban landscape surrounding the fragments (see also [70]). Such a scenario would suggest a possible opportunity for enhanced urban habitat management to lessen the effects of extreme climate events in ecosystems fragmented by urban development [71,72]. An alternative, but not mutually exclusive, explanation is that reserve sites might have harbored a greater proportion of narrowly adapted species that are sensitive to various forms of environmental perturbation, whereas ecological filtering might have already eliminated sensitive species from our habitat fragments [22], such that further disturbance in the form of the drought event in 2014 might have had lesser impacts on bee diversity and functional composition in fragments. In this latter case, our finding of lesser impacts of drought in fragments may be evidence that there is a core set of functional trait combinations that confer resistance to both habitat fragmentation and drought. This finding also suggests that the processes that we hypothesized would maintain bee diversity and functional trait evenness in reserves—i.e., higher taxonomic and functional diversity of bee assemblages and greater connectivity among microhabitats in the landscape—did not result in improved responses of reserve bee assemblages to drought, at least in the timeframe of our study.

Our finding of increased proportional representation of eusocial species and increased bee abundance led us to investigate whether these phenomena were driven by the dominant eusocial bees in our system—*Lasioglossum* subgenus *Dialictus*, a cosmopolitan, small-bodied, primarily eusocial group known to reach high abundances in many systems, including other xeric regions [73,74,75] and human-modified habitats [25,70,76]. Indeed, the substantial increase in both relative and absolute abundance of *Dialictus* species post-drought suggests that species in this group are ecological “winners” [43] with respect to their ability to persist through drought events. The success of this group may be attributed to their small size and generalist diet, given that these traits would allow *Dialictus* species to subsist on low quantities of food resources from any plant species in the environment that may bloom during the drought, including weedy species in anthropogenic landscapes [77]. In support of this postulation, the abundance of other small-bodied pollen generalists (intertegular distance < 1.4 mm; consisting mostly of non-eusocial *Ceratina* spp. and primitively eusocial *Lasioglossum* (*Sphecodogastra*) *nigrescens* (Crawford) and *Halictus tripartitus* Cockerell) also did not decrease after the drought (Poisson GLMM *z* = 1.46, *p* = 0.14; no significant interaction between drought and fragmentation), in contrast to the post-drought declines exhibited by the rest of the non-*Dialictus* bee assemblage (Figure 4c). It is worth noting, however, that even among *Dialictus* species, there were significant turnovers in relative abundance in response to drought (Figure S4a, Table S4). These turnovers indicate that not all *Dialictus* species were similarly capable of taking advantage of drought conditions, and underscore the pressing need to fill gaps in our understanding of the natural history of *Dialictus* species in our bioregion, including their alpha taxonomy (see Table S2).

The increased abundance of *Dialictus* drove many of our findings, as revealed by a set of analyses on only the non-*Dialictus* species in our assemblages (Figure 4, Figure S4). The decrease in non-*Dialictus* bee abundance suggests that the drought event indeed resulted in reduced persistence (or at least, activity) of the overall bee fauna in the following years. The absence of pre- versus post-drought differences in the mean body size of non-*Dialictus* bees perhaps suggests that neither mobility nor resource requirements are chief drivers of bee species’ response to drought events, unlike their role in modifying the response of bees to habitat fragmentation [78]. Similarly, the lack of pre- versus post-drought differences in the proportional representation of pollen generalists among non-*Dialictus* bees suggests that specialists may not be disproportionately impacted by drought. Pollen specialists may withstand drought by associating with drought-hardy plant species, or by evolving more finely-tuned synchronization of phenologies with their host plants, including greater propensity to aestivate through drought years when environmental conditions prohibit their host plants from blooming [19,26] (but see [18]). Lastly, decreases in the relative abundance of non-*Dialictus* eusocial bees in fragments after the drought event suggest that for the rest of the eusocial taxa, heightened requirements for ample and consistently available food resources [27,28] may have imposed a fitness cost that could not be overcome by a generalist diet. Contrasting responses of different eusocial bee taxa to drought (if indeed no *Dialictus* species in our system exhibits solitary life history atypical for the subgenus) indicate that a nuanced approach must be taken when attempting to understand and predict the response of bees to environmental perturbations based on examinations of single functional traits.

Despite the many instances of significant interactions in univariate analyses demonstrating that fragment sites were less impacted by drought than were reserve sites, our multivariate analyses did not reveal such an interaction. Instead, the impacts of habitat fragmentation and drought appeared to additively alter the composition of bee assemblages. This finding may be driven by turnovers of species within individual functional groups (e.g., the case of *Dialictus* species, Figure S4a) in response to drought; such turnovers may override the differential changes between reserves and fragments in the relative abundances of different functional groups detected in univariate analyses. Significant interannual turnover of bee assemblages is a well-known phenomenon [60,73,74] and should be expected in our study system as well. However, the strong differentiation between bee assemblages in pre- versus post-drought years, in stark contrast to the close correspondence between bee assemblages in the two pre-drought years and two post-drought years, implicates the drought event as a driver of this shift in composition. In fact, bee assemblage composition differed more between pre- and post-drought years than between reserves and fragments in any one year, such that the *R^2^* value of study year is nearly 3.5 fold higher than that of fragmentation status (Figure 5). This finding suggests that extreme climate events may have stronger impacts on bee assemblages compared to local-scale anthropogenic habitat modification (see also [79,80]). On the other hand, we found no difference across study years or between reserves and fragments in the compositional dispersion (i.e., beta diversity) of bee assemblages. This lack of difference in dispersion suggests that neither disruptive force imposed a sufficiently strong filter to result in the domination of a small subset of bee species across study sites (i.e., biotic homogenization [81]).

The chief limitations of our study are its short duration and, in particular, a lack of bee surveys conducted during the 2014 drought event. Although comparisons of bee assemblages before and after the drought event shed light on changes in the species and functional trait compositions of bee faunas in response to drought, such comparisons provide no inference regarding bee diversity, abundance, phenology, and interactions with plants during the drought event itself. Additionally, lack of sampling during the drought event means that we are unable to distinguish between bee species that successfully reproduced during the drought and those that were able to avoid the impacts of drought through multiple-year dormancy. The ability of bees to remain dormant during years of poor conditions is well known for species that occur in desert ecosystems [19,26,82], but less so for species in Mediterranean-climate areas like coastal southern California. Given that bee assemblages in our CSS system likely include both species that can remain dormant (e.g., many *Diadasia* species that also occur in adjacent deserts) and those that cannot (e.g., *Bombus*, and perhaps other mesic-adapted taxa), our results should be interpreted with caution. Future data collection during drought events in our system is needed to fill the knowledge gap regarding the identities of bee species that can remain dormant to avoid droughts, and, in the event of multi-year droughts, the number of consecutive years such species may remain dormant. Such knowledge may be critically necessary for proper interpretation of data collected in large-scale efforts to monitor bee populations (e.g., [83]), as the detected bee assemblage in flight may not reflect the true viable population of both active and dormant bees, especially in temperate xeric habitats in which the majority of the world’s bee biodiversity is concentrated [84]. We also caution that our results are generalizable only to the first two years of bee assemblage recovery after a drought event. Reserves and fragments, which were indistinguishable from each other in post-drought comparisons with respect to most of the metrics we investigated, may prove to have divergent recovery trajectories with additional years of monitoring post-drought. In the same vein, given that our study focuses on a single drought event, data on additional events will be necessary to demonstrate whether or not the patterns we report are generalizable to droughts in general in our system, or if each drought event causes idiosyncratic and unpredictable shifts in bee assemblages. Lastly, our use of bowl traps also constrains the resolution and generality of our findings, especially since we did not consistently collect plant assemblage data that would enable investigating the impact of floral abundance in the surrounding area on bowl trap capture rates (see [49]). However, the divergent numerical responses of *Dialictus* versus non-*Dialictus* bees (Figure 4b,c) seem to provide indirect evidence that differences in floral abundance pre- versus post-drought (if any such difference did exist) did not drive our key findings, since one would expect impacts of floral abundance on capture rates to act similarly on the entire bee assemblage.

## 5. Conclusions

The severe drought event in 2014 had profound impacts on bee assemblages that were, in many cases, more acute than the impacts of habitat loss and fragmentation. Thus, future conservation efforts aimed at preserving at-risk bee populations and maintaining available pollination services may need to focus on mitigating the impacts of climate change at least as much as those of habitat loss. More long-term monitoring of bee assemblages spanning drought events of differing durations will be necessary to fully appreciate the potentially diverse responses of different bee species to the challenges brought about by drought and its impacts on the biotic and abiotic environment. The ability to better predict shifts in the activity and population dynamics of bee assemblages in response to extreme climate events will be helpful in guiding efforts to conserve the bee taxa that are most sensitive to the joint impacts of drought and habitat fragmentation in our system and other hotspots of bee diversity threatened by similar pressures.

## Figures and Tables

**Table 1:** Results of post-hoc PERMANOVA analyses comparing bee assemblage composition across all possible pairs of study years in both pre-drought (2011 and 2012, “Pre”) and post-drought (2015 and 2016, “Post”) conditions. Reported *p* values have been adjusted using Benjamini-Hochberg false discovery rate (FDR) correction for multiple comparisons.

Comparison	Type	Test Statistic	*R* ^2^	*p* Value
2011 versus 2015	Pre-Post	*F*_1,17_ = 7.88	0.29	0.0015
2011 versus 2016	Pre-Post	*F*_1,17_ = 5.60	0.23	0.0015
2012 versus 2015	Pre-Post	*F*_1,26_ = 11.23	0.28	0.0015
2012 versus 2016	Pre-Post	*F*_1,26_ = 7.87	0.21	0.0015
2011 versus 2012	Pre-Pre	*F*_1,22_ = 2.54	0.09	0.011
2015 versus 2016	Post-Post	*F*_1,21_ = 1.70	0.07	0.083

## Data Availability

Bee specimen data presented in this study are openly available in the UC San Diego Library Digital Collections: data from 2011–2012 are available at https://doi.org/10.6075/J000001W (reference number bb3415450r), and data from 2015–2016 are available at https://doi.org/10.6075/J069724P (reference number bb3180261d). Information on study plots and life history traits of bee species are available in Supplementary Tables S1 and S2, respectively.

## Supplementary Materials

The following are available online at https://www.mdpi.com/2075-4450/12/2/135/s1. Supplementary Tables—Table S1: Study plots included in this study, Table S2: List of bee species and their life history traits, Table S3: Statistical details pertaining to post-hoc pairwise comparisons of treatment combinations in univariate analyses presented in Figure 2, Figure 3 and Figure 4, Table S4: Statistical details pertaining to post-hoc pairwise comparisons among study years in multivariate analyses presented in Figure S4. Supplementary Figures—Figure S1: Qualitative results of auxiliary univariate analyses corresponding to those presented in Figure 3 and Figure 4 retaining only study plots sampled both before and after the drought, Figure S2: Qualitative result of auxiliary multivariate analysis corresponding to that presented in Figure 5 retaining only study plots sampled both before and after the drought, Figure S3: Results of auxiliary univariate analyses corresponding to those presented in Figure 3 and Figure 4 using study year instead of drought status as a main effect, Figure S4: Results of auxiliary multivariate analyses to examine the contribution of *Lasioglossum* subgenus *Dialictus* to changes in bee assemblage composition in response to drought and habitat fragmentation.
