# Supplementary material for: Joint Impacts of Drought and Habitat Fragmentation on Native Bee Assemblages in a California Biodiversity Hotspot
Source: Insects. 2021 Feb 5;12(2):135. doi: 10.3390/insects12020135 (PMC7914906; doi:10.3390/insects12020135)
Supplement: Supplementary file 1 [file insects-12-00135-s001.zip › Supplementary Materials/Supplementary Figures.docx]

**
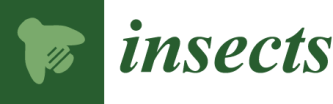

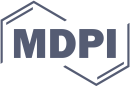
**

Article

Joint impacts of drought and habitat fragmentation on native bee assemblages in a California biodiversity hotspot

Keng-Lou James Hung^1,2,*^, Sara S. Sandoval^1^, John S. Ascher^3^ and David A. Holway^1^

^1^ Section of Ecology, Behavior and Evolution, Division of Biological Sciences, University of California - San Diego, La Jolla, CA 92037, USA.

^2^ Department of Ecology and Evolutionary Biology, University of Toronto, Toronto, Ontario M5S 3B2, Canada.

^3^ Department of Biological Sciences, National University of Singapore, 14 Science Drive 4, Singapore 117543, Singapore.

* Correspondence: [kenglou.hung@gmail.com](mailto:kenglou.hung@gmail.com)

**Supplementary Figures S1 – S4**


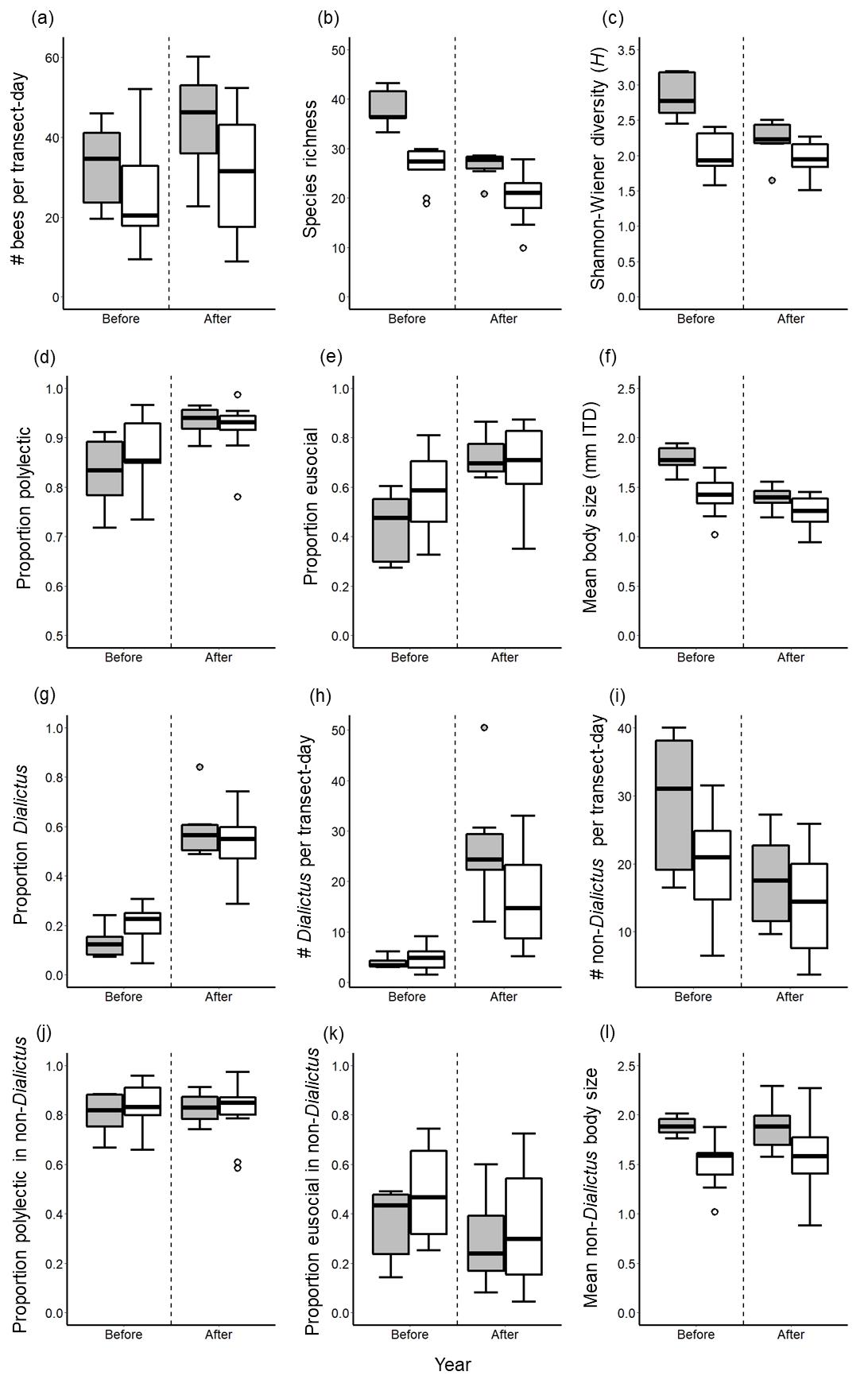


**Figure S1.** Caption on next page.

**Figure S1, continued from previous page.** Comparisons of bee assemblages in a subset of scrub reserve plots (gray boxes) and scrub fragment plots (white boxes) that were sampled both before (2011 and 2012) and after (2015 and 2016) a severe drought event in 2014. Models are constructed as in Figures 3 and 4 of the main text, but with the exclusion of data from plots sampled only before or only after the drought. Boxplots are as in Figures 3 and 4, where panels (a) through (f) correspond to the analyses reported in Figure 3a through 3f and panels (g) through (l) correspond to those in Figure 4a through 4f, respectively. Due to the qualitative nature of this auxiliary analysis, no formal statistical comparisons were performed.


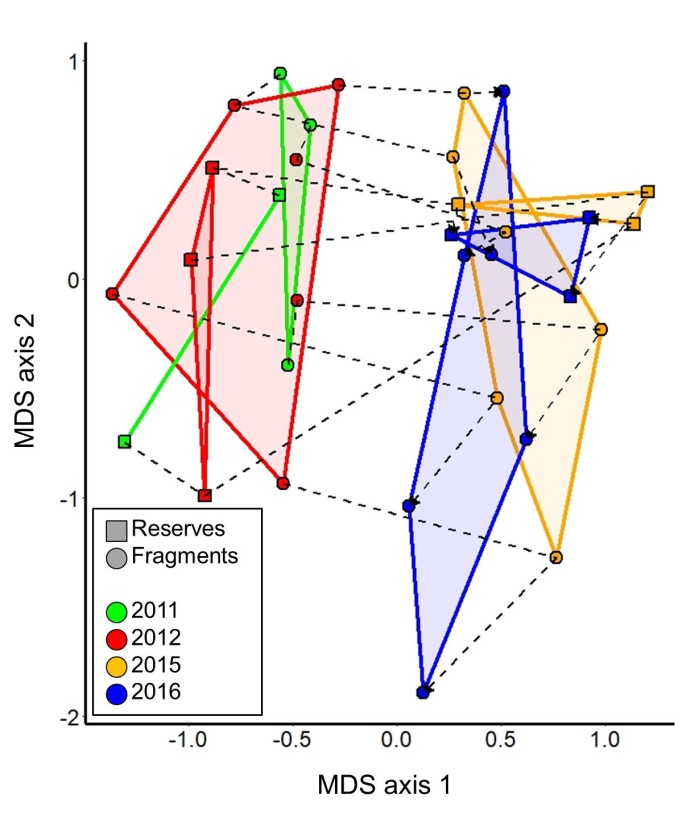


**Figure S2.** Nonmetric multidimensional scaling (NMDS) ordination plot of bee assemblages in a subset of reserve plots (squares) and fragment plots (circles) that were sampled both before (2011 and 2012) and after (2015 and 2016) a severe drought event in 2014. Points depicting sequential years of sampling at the same study site are connected by dotted lines. The ordination was constructed based on Bray-Curtis dissimilarity scores between all possible pairs of plot-year combinations. Dissimilarity scores were calculated based on relative abundances of bee species after standardizing each assemblage to a sum of 1. Due to the qualitative nature of this auxiliary analysis, no formal statistical comparisons were performed.

**
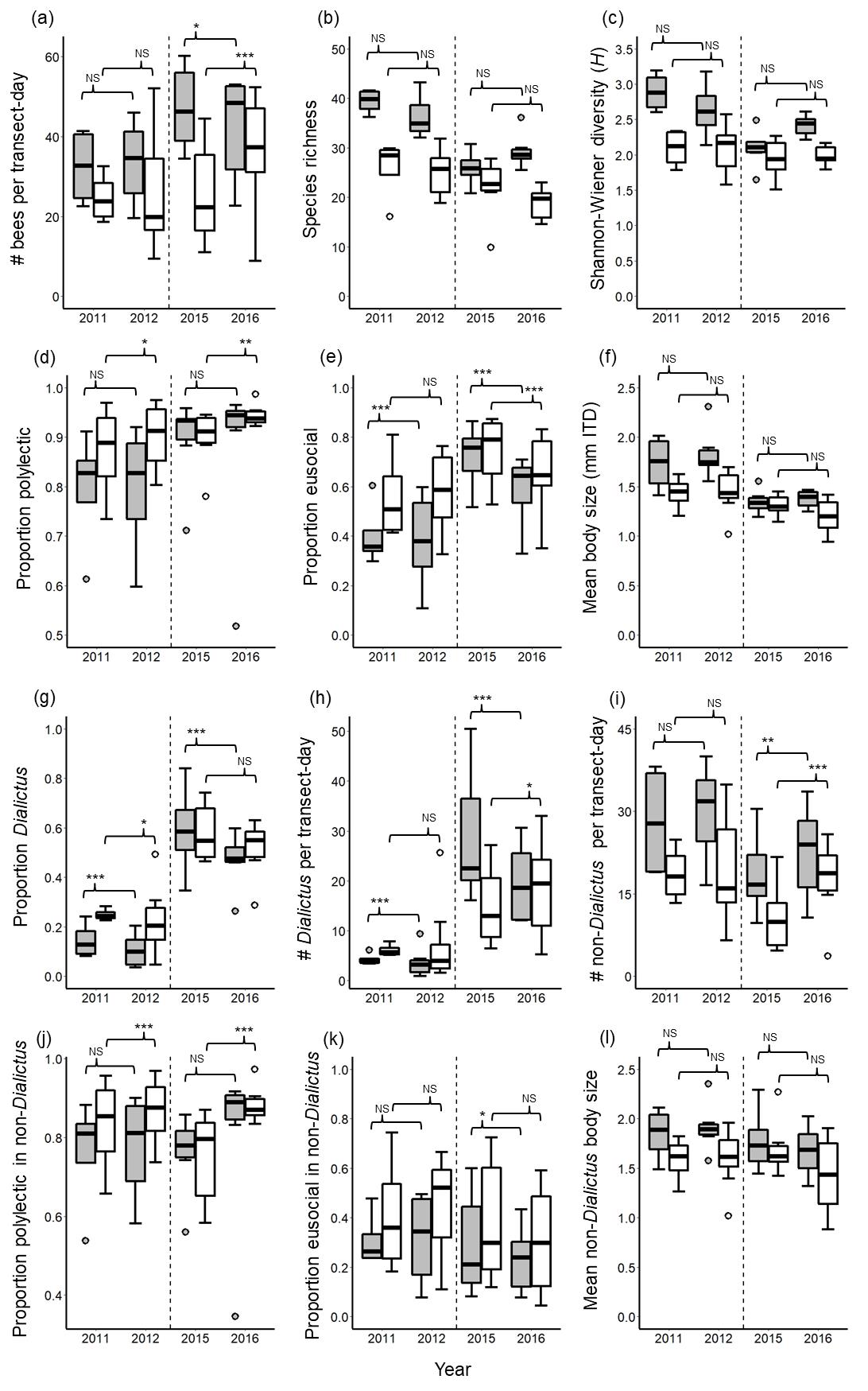
**

**Figure S3.** Caption on next page.

**Figure S3, continued from previous page.** Comparisons of bee assemblages in scrub reserves (gray boxes) and scrub fragments (white boxes) across four study years that spanned a severe drought event in 2014 (dotted line). Models are constructed as in Figures 3 and 4 of the main text, but with study year as a main effect in place of drought status (before vs. after drought). Boxplots are as in Figures 3 and 4, where panels (a) through (f) correspond to the analyses reported in Figure 3a through 3f and panels (g) through (l) correspond to those in Figure 4a through 4f, respectively. Statistical significance of comparisons between years in the same drought status is provided for reserves and fragments separately: n.s. *P* > 0.05, * *P* ≤ 0.05, ** *P* ≤ 0.01, *** *P* ≤ 0.001.


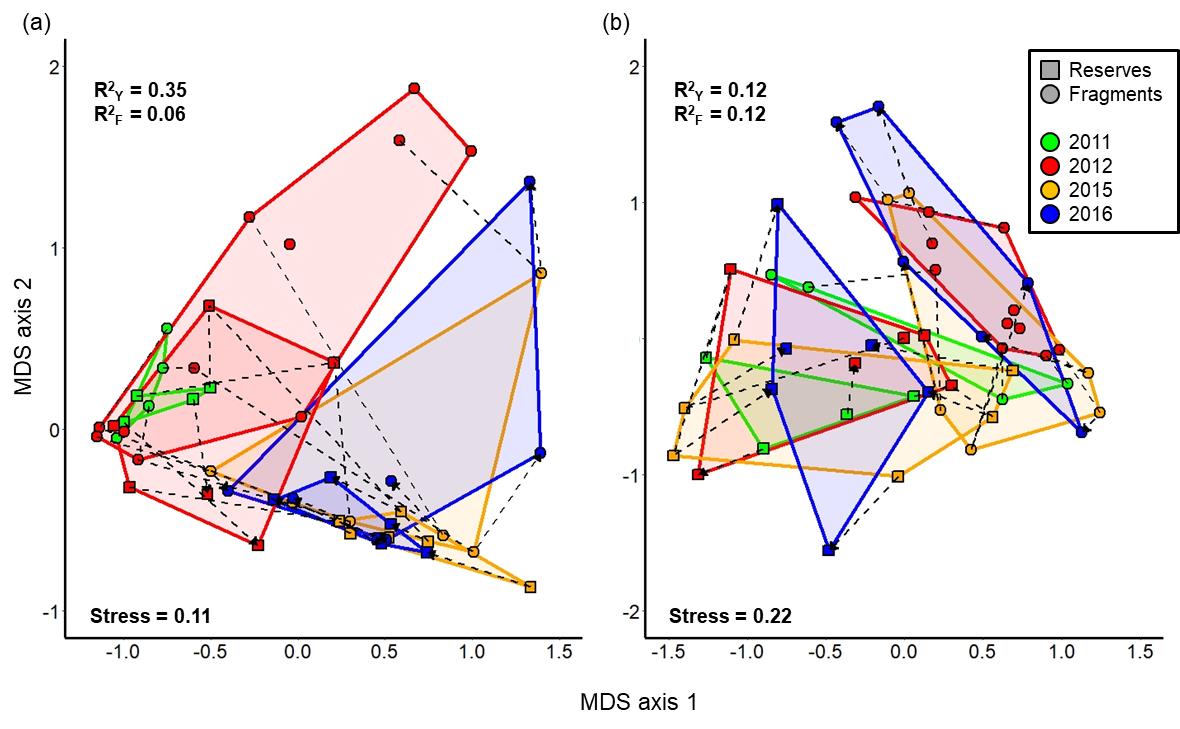


**Figure S4.** Nonmetric multidimensional scaling (NMDS) ordination plot of bee assemblages in reserve plots (squares) and fragment plots (circles) across four years of sampling that spanned a severe drought event in 2014. Panel (a) depicts an ordination that includes only individuals of *Lasioglossum* subgenus *Dialictus*, panel (b) depicts an ordination with all *Dialictus* individuals excluded from the analysis. Points depicting sequential years of sampling at the same study site are connected by dotted lines. The ordination was constructed based on Bray-Curtis dissimilarity scores between all possible pairs of plot-year combinations. Dissimilarity scores were calculated based on relative abundances of bee species after standardizing each assemblage to a sum of 1. *R^2^* values are indicated for each PERMANOVA independent variable: Y = study year, F = fragmentation status (reserves vs. fragments). Bee assemblage composition differed significantly across study years in both panel (a) (*F*_3,41_ = 8.65, *P* < 0.001) and panel (b) (*F*_3,41_ = 2.37, *P* < 0.001). Bee assemblage composition also differed across reserves and fragments in both panel (a) (*F*_1,41_ = 6.82, *P* < 0.001) and panel (b) (*F*_1,41_ = 4.19, *P* = 0.012). There was no statistically significant interaction between drought status and fragmentation status in either analysis. Detailed statistical outputs of post-hoc pairwise comparisons between all possible pairs of study years are reported in Table S4.
